# Impaired oligodendroglial turnover is associated with myelin pathology in focal cortical dysplasia and tuberous sclerosis complex

**DOI:** 10.1111/bpa.12452

**Published:** 2017-02-09

**Authors:** Theresa Scholl, Angelika Mühlebner, Gerda Ricken, Victoria Gruber, Anna Fabing, Sharon Samueli, Gudrun Gröppel, Christian Dorfer, Thomas Czech, Johannes A. Hainfellner, Avanita S. Prabowo, Roy J. Reinten, Lisette Hoogendijk, Jasper J. Anink, Eleonora Aronica, Martha Feucht

**Affiliations:** ^1^ Department of Pediatrics and Adolescent Medicine Medical University of Vienna Vienna Austria; ^2^ Institute of Neurology, Medical University of Vienna Vienna Austria; ^3^ Department of Neurosurgery Medical University of Vienna Vienna Austria; ^4^ Department of (Neuro) Pathology Academic Medical Centre Amsterdam The Netherlands; ^5^ Swammerdam Institute for Life Sciences, Centre for Neuroscience, University of Amsterdam Amsterdam The Netherlands; ^6^ Stichting Epilepsie Instellingen Nederland (SEIN) Heemstede The Netherlands

**Keywords:** epilepsy, region of interest (ROI) based image analysis, oligodendroglial hyperplasia

## Abstract

Conventional antiepileptic drugs suppress the excessive firing of neurons during seizures. In drug‐resistant patients, treatment failure indicates an alternative important epileptogenic trigger. Two epilepsy‐associated pathologies show myelin deficiencies in seizure‐related brain regions: Focal Cortical Dysplasia IIB (FCD) and cortical tubers in Tuberous Sclerosis Complex (TSC). Studies uncovering white matter‐pathology mechanisms are therefore urgently needed to gain more insight into epileptogenesis, the propensity to maintain seizures, and their associated comorbidities such as cognitive defects. We analyzed epilepsy surgery specimens of FCD IIB (*n* = 22), TSC (*n* = 8), and other malformations of cortical development MCD (*n* = 12), and compared them to autopsy and biopsy cases (*n* = 15). The entire lesional pathology was assessed using digital immunohistochemistry, immunofluorescence and western blotting for oligodendroglial lineage, myelin and mTOR markers, and findings were correlated to clinical parameters. White matter pathology with depleted myelin and oligodendroglia were found in 50% of FCD IIB and 62% of TSC cases. Other MCDs had either a normal content or even showed reactive oligodendrolial hyperplasia. Furthermore, myelin deficiency was associated with increased mTOR expression and the lower amount of oligodendroglia was linked with their precursor cells (PDGFRa). The relative duration of epilepsy (normalized to age) also correlated positively to mTOR activation and negatively to myelination. Decreased content of oligodendroglia and missing precursor cells indicated insufficient oligodendroglial development, probably mediated by mTOR, which may ultimately lead to severe myelin loss. In terms of disease management, an early and targeted treatment could restore normal myelin development and, therefore, alter seizure threshold and improve cognitive outcome.

## Introduction

Affecting approximately 50 million people, epilepsy is one of the most common neurological diseases worldwide (WHO). Although about two‐third of all epilepsy patients respond well to medical treatment with antiepileptic drugs (AEDs), 30%–40% cases are, or become, drug‐resistant 18, 19. For about one third of these patients, epilepsy surgery is an effective alternative treatment option 2. For those who are not surgical candidates, including patients with impaired cortical development and associated myelin deficiencies, new treatment strategies are urgently needed and, therefore, studies attempting to uncover the molecular basis of epileptic seizures and epileptogenesis or detect key targets for new therapies are of crucial importance.

Malformations of cortical development (MCDs) are the most common pathologies found after epilepsy surgery in patients younger than 18 years of age 15. The majority of these lesions are Focal Cortical Dysplasias (FCDs) 38. FCDs are currently diagnosed according to the International League Against Epilepsy (ILAE) classification scheme introduced in 2011 3. This classification system distinguishes three FCD categories: Type I describes isolated focal lesions with architectural abnormalities, type II includes isolated focal lesions with architectural abnormalities and aberrant cell forms, and type III refers to cortical disorganization associated with—or adjacent to—other principal lesions 3. Another disease increasingly treated by epilepsy surgery is Tuberous Sclerosis Complex (TSC). It is a rare genetic disorder with an estimated prevalence of between 1:6800 and 1:15000 newborns 26, 47. Mutations in two genes, *TSC1* and *TSC2*, are responsible for pathological constitutive activation of the highly conserved PI3K‐mTOR pathway 20. These gene mutations lead to the development of benign tumors in almost all organ systems, among them subependymal giant cell astrocytoma, cortical tubers, and neuronal migration lines (NMLs) in the brain.

Over the past decades, efforts have been made to uncover the underlying pathogenesis of FCD. Only very recently, somatic (mosaic) mutations in several genes also regulating the mTOR pathway have been identified for FCD IIA and IIB, including: PTEN; PI3KCA; AKT 17, 33; the DEPDC5 and NPRL3 components of the mTOR regulatory GATOR‐1 complex 4, 36; as well as mTOR itself 8, 21, 25. Therefore, it seems logical that these lesions have common morphological features. In addition to a thickened cortex and a blurred gray‐white matter boundary, the other most prominent feature in TSC as well as in FCD IIB (detectable using pre‐surgical MRI) is the white‐matter deficiency. This is usually referred to as “transmantle sign” 44.

In general, myelinogenesis is defined by the establishment of myelin sheaths and is critical for the optimization of conduction velocity, maturation, survival, and regenerative capacity of axons 27. The cells responsible for this process are called oligodendrocytes that mature from precursor cells termed oligodendrocyte progenitor cells (OPCs) 48. The different stages undertaken by OPCs to become mature oligodendrocytes can be assessed using several markers. For example, juvenile OPCs express high amounts of PDGFRa 42, later additional NG2 41; intermediate stages express mainly O4 39; whereas fully mature oligodendrocytes express CNPase, NogoA, and MBP 35 and the older oligodendrocytes express Tppp 16, 48. Mature cells have the capability to renew their myelin sheaths three times within 24 h 22, 28. In its lifetime, a single oligodendrocyte is able to enwrap axons of up to 50 neurons 14, 22, 28.

Impairment of this fragile system can have a high impact on myelination and may—in the worst case—lead to distorted or interrupted neurotransmissions 6. The most prominent disorder linked to myelin deficiencies is multiple sclerosis (MS) 9, but abnormalities are also seen in autism and schizophrenia 13. It is still unclear if the observed myelin pathology in epilepsy surgery specimens is primarily associated with the underlying malformative process, or is just a secondary phenomenon of ongoing epileptic seizures. Recent studies identified an association between myelin reduction and a lower number of oligodendrocytes, supporting the hypothesis that myelin pathology is the primary reason for seizures 23, 49. However, results remain controversial: while Shepherd *et al* indicated a positive correlation between white matter reduction and the duration of epilepsy 35, studies performed on patients with temporal lobe epilepsy and mild malformations of cortical development showed an increased amount of oligodendroglia compared to controls 34, 40.

Here, we investigated the mechanisms associated with reduced myelination in patients with epilepsy because of FCD‐ and TSC‐associated tubers. Using immunohistochemistry, we inspected the myelination of axons as well as the content of oligodendroglia cells. Immunofluorescence and western blotting were employed to study specific markers for proliferation and myelination of oligodendroglial lineage.

## Materials and Methods

### Inclusion criteria and group characteristics

We reviewed pediatric patients (range 0–18 years) with MCD and drug‐resistant epilepsy who underwent epilepsy surgery at the Pediatric Epilepsy Center of the Medical University Vienna. A re‐evaluation of corresponding formalin fixed and paraffin embedded (FFPE) resected brain material from the Neurobiobank of the Institute of Neurology was performed according to the new classification scheme (3). Each of these individual tissue blocks had to contain sufficient white matter. Cortical tubers from patients with TSC, FCDs and mild malformations of cortical development (MMCD) were included. Five TSC cases were included from the Neurobiobank AMC Amsterdam. Age‐ and region‐matched white matter tissue of autopsy and biopsy samples (without infiltration of tumor cells on histology) served as control group. Additional, all the cases of the control group were not allowed to show a history of epilepsy or any other neurologic disease. Besides the histological diagnosis, clinical data like gender, age, seizure frequency, relative epilepsy duration (normalized to age), AEDs, post‐surgical outcome 45 and location of resected material was assessed. The study was performed according to the guidelines of good laboratory practice of the European commission, and the local ethics committee of the Medical University of Vienna gave a positive vote for the study plan (EC978/2009).

### Sequencing

Somatic mutation analysis was performed with FFPE brain lesion material. For NGS, the Ion AmpliSeq™ Neurology Panel (ThermoFisher Scientific, Waltham, Massachusetts) was used for targeted multi‐gene amplification of: AKT1, AKT3, ATRX, BRAF, CDK6, CIC, CTNNB1, DDX3X, DEPDC5, EZH2, FGFR1, FUBP1, H3F3A, HIST1H3b, HIST1H3c, IDH1, IDH2, KDM6A, mTOR, MYB, MYBL1, NPRL2, NPRL3, PIK3CA, PIK3R1, PIK3R2, PTCH1, PTEN, SMARCA4, SMARCB1, SMO, SUFU, TP53. The Ion AmpliSeq Library Kit 2.0 was used for libraries and Ion PGM Hi‐Q Kit and Ion Chef Instrument for emulsion PCR and template preparation. The Ion PGM Hi‐Q sequencing Kit was used with the Ion 318 V2 Chip and the sequencing platform was the Personal Genome Machine. About 5% was chose as background noise to determine the total number of existing non‐synonymous driver‐gene mutations per lesion. DNA from blood samples were used for single gene analysis of TSC1 and/or TSC2 were performed with Sanger Sequencing. Unknown alterations were compared with MutationT@, PolyPhen2 and 1000Genomes Database.

### Immunohistochemistry

After surgery the specimens were routinely processed for histopathology. The antibodies and dilutions (Table 1) were used on 3‐µm tissue sections, which were mounted on negatively charged glass slides. The EnVision™ FLEX+ kit (Dako, Glostrup, Denmark) was used as a detection system and diaminobenzidine (DAB) as chromogen and either processed with an autostainer (Dako, Glostrup, Denmark) or via coverplates (Thermo Scientific, Glass Coverplates). Sections were counterstained with hematoxylin.

**Table 1:** Antibodies and dilutions for standardized immunohistochemical staining procedure.

**Antibody overview**						
Antibody	Company	Clone	Dilution	Pretreatment	Method	Incubation
CD3	Neomarker	SP7	1:200	pH6 citrate buffer	Autostainer	30 min room temp
CNPase	Millipore	11‐5B	1:500	pH6 citrate buffer	Coverplates	Over night 4°C
MAP2	Millipore	AP20	1:8000	pH6 citrate buffer	Autostainer	30 min room temp
MBP	Dako	A0623	1:400	pH6 citrate buffer	Autostainer	30 min room temp
MOBP	Sigma	HPA035152	1:100	pH9	Coverplates	Over night 4°C
NeuN	Millipore	A60	1:2000	pH6 citrate buffer	Autostainer	30 min room temp
NG2	Millipore	AB5320	1:200	non	Humidity chamber	Over night 4°C
NogoA	Millipore	AB5888	1:1000	pH6 citrate buffer	Coverplates	Over night 4°C
Olig2	IBL	18953	1:100	pH6 citrate buffer	Autostainer	60 min room temp
PDGFRa	Abcam	ab61219	1:50	non	Humidity chamber	Over night 4°C
pS6(Ser240/244)	Cell Signaling	D68F8	1:1000	pH6 citrate buffer	Coverplates	Over night 4°C
SMI32	Covance	SMI‐32R	1:200	pH6 citrate buffer	Autostainer	30 min room temp
Tppp7/p25	Clin. Center, Univ. Pecs	mAb6C1	1:1000	pH6 citrate buffer	Autostainer	30 min room temp
Vimentin	Dako	V9	1:50	non	Autostainer	30 min room temp

### Immunofluorescence

Double staining was performed via immunofluorescence standard procedure. In brief, primary antibodies were incubated with pre‐treated (compare IHC Table 1) FFPE tissue sections for two hours at room temperature. Secondary antibodies (AF488 A‐11029 Thermo Scientific, Cy3 016‐160‐084 Jackson ImmunoResearch) were incubated for one hour in the dark. Nucleus staining for orientation was done with DAPI mounting medium (VECTASHIELD, H‐1200 Vector Laboratories). Fluorescence microscopy and image overlay was performed with Zeiss Axio Imager Z1 microscope and Ikaros. & Isis. (Version 5.1) software.

### ROI‐based approach for slide analysis

**Figure 1:**
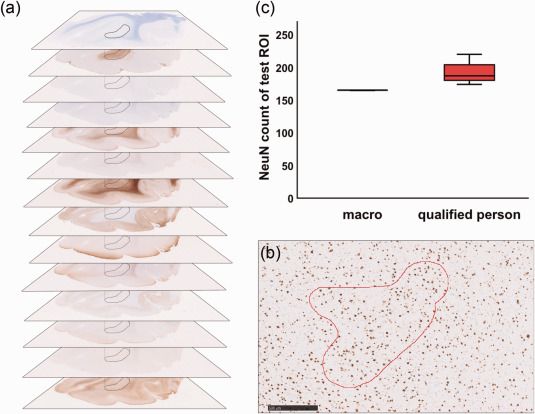
Macro validation. **a** Immunohistochemical digital slide stack, black = region of interest (ROI), kept constant during individual stainings. **b** NeuN staining, red = test ROI for macro validation, scale bar = 500 µm. **c** Statistical analysis of macro validation. 10 individual macro runs were compared to counts of 10 individual qualified persons.

In order to generate sufficient data for statistical analysis and to detect associations between abnormal myelination and pathology sub‐types, we tested the suitability of a novel approach that focuses on the region of interest (ROI) in a particular slide. Approximately 15 slides of each brain specimen were used, each stained for a different marker. Using an especially written algorithm, the number of cells and the intensity of signals of the whole pathologic region were analyzed (Figure 1). The slides were scanned at 400× magnification and each digitalized staining was visually inspected, with the NDPview software (Hamamatsu, NanoZoomer), converted to .tiff, and then transferred to ImageJ (Version 64, open source). Selected white matter regions of interest were delineated on the basis of myelin (Nissl‐LFB), balloon/giant cells (vimentin) and (dysmorphic) neurons (SMI32/MAP2) (Figure 1a). For each antibody, an optimal threshold, which distinguished between positive and background staining, was evaluated. These were as follows: CD3 0‐228, CD68 0‐217, CNPase 0‐165, MBP 0‐115, MOBP 0‐195, NeuN 0‐200, NogoA 0‐218, Olig2 0‐215, PDGFRa 0‐222, pS6 0‐210, Tppp 174‐190, vimentin 0‐111. Because of variable staining intensities within the Olig2 autopsy cases (caused by non‐standardized post‐mortem fixation times), a case dependent threshold level was selected individually. In order to validate the method, a slide was presented to ten qualified pathologists and technicians who manually evaluated the same data, and the results were compared (Figure 1b). The data obtained electronically was superior to that collected manually since the program consistently counted the signals from the same region, whereas data collected by our qualified personnel was more variable (Figure 1c).

### Semiquantitative assessment of oligodendroglial proliferation

For determination of oligodendroglial proliferation (Ki67 and Olig2/Ki67) at least 100 nuclei were counted at high magnification (400×) and the average number of (double) positive cells was expressed as percentage 1.

### Western blotting

Western blotting was done to determine oligodendroglial lineage protein levels (NG2, Olig2, MBP) and to subsequently detect differences between epilepsy patient groups. It could only be performed in eight cases of which frozen material was available. Material from seven patients (MUV; MMCD: *n* = 2; age = 9, 13a/FCD IIB: *n* = 3; age = 8, 17, 16a/TSC: *n* = 2; age = 3, 10a), and one autopsy control (AMC; age = 4a) was analyzed. White matter was lysed with RIPA lysis buffer (Upstate, 20‐188) plus phosphatase 2,3 (Sigma‐Aldrich, P5726, P0044) and protease inhibitor cocktail (Sigma‐Aldrich, P8340). After measuring protein concentration via Lowry assay, 15μg of protein was mixed with sample buffer (2× Laemmli sample buffer, BIO‐RAD), loaded on SDS‐gels (Mini‐Protean TGX Precast Gels; 4%–12%, Bio‐Rad) and run for 80 min at 100 V. Tank blotting occurred with PVDF membranes at 100 V for 60 min. For the next step, membranes were washed with 3% milk for one hour to block unspecific bindings. Primary antibodies (ßActin 1:50 000 Sigma Aldrich, A3854, MBP 1:20000, NG2 1:300, Olig2 1:50, compare Table 1) were then applied to membranes overnight. After washing steps with 1xTBST, secondary antibodies (P044701 and P044801, DAKO) were applied for 1 h. Developing of membranes occurred with ECL solution (Amersham ECL Prime Western Blotting detection reagent). Chemiluminescence detection was performed via BioRad ChmeiDoc XRS Quantity One system.

### Statistical analysis

Graphical data visualization was accomplished in GraphPad Prism (Version 7) and SPSS (Version 23.0). Statistical analysis was performed in SPSS (IBM software). Non‐parametric independent Kruskal–Wallis and all‐pairwise comparisons were used to analyze differences within groups. Kendall‐tau b was undertaken for correlations. Categorical data was compared utilizing Chi‐square. Unsupervised hierarchical clustering was performed to determine subgroups (Ward's method, squared Euclidian distances). Not significant values are indicated by n.s., *P* < 0.05 was considered to be significant and was indicated by stars (*).

#### Declaration

At the Medical University of Vienna (MUV) IHC/IF stainings, western blotting, data analysis and manuscript preparation were done. At the Academic Medical Centre (AMC, Amsterdam) sequencing, statistical counseling and project guidance were performed. Additional, AMC also provided material of five TSC patients and two controls.

## Results

### Subjects (and material)

In total, we included material from 42 patients: eight cortical tubers from patients with TSC, 22 FCD IIB, six FCD IIA and six MMCD. The control group consisted of ten autopsy samples and five biopsy samples adjacent to a surgically removed tumor (Table 2). Mutations within mTOR signaling were seen in all TSC (one TSC1 and seven TSC2) and some in FCD IIB samples (one SMO, one NPRL3, and one SUFU; Table 2).

**Table 2:** Patient data overview. *N* refers to number of cases. Seizure frequency was split in daily and weekly, unit of age (mean)—years and relative duration (mean) was normalized to age in %. Cognition was split in: n = normal, m = mild impairment, i = intermediate impairment, s = severe impairment. Location of resected tissue: c= central, f = frontal, h = hemispheric, o = occipital, p = parietal, t = temporal. * indicates missing data.

Clinical data								
Type	*n*	Sex (F:M)	Age (±SD)	Frequency d/w	Duration (±SD)	Mutations	Cognition	Location
Autopsy and biopsy	15	8:7	13 (6,3)	–	–	–	15n	3f/4t/1o/1p*
TSC	8	3:5	9 (7,1)	5/3	78 (18)	1TSC1/7TSC2	2n/2m/1i/3s	5f/3t
MMCD	6	1:5	11 (3,6)	3/3	65 (21)	–	2m/1i/3s	3f/2t/1p
FCD IIA	6	2:4	8 (4,9)	3/3	65 (20)	–	2n/3m*	2f/1t/2o/1h
FCD IIB	22	9:13	9 (11,8)	11*/7*	68 (18)	1SMO/1NPRL3 1SUFU	8n/3m/4i/2s *	10f/3t/2o/6c/1p
Total	57	23:34	10 (8,7)	22/16	51 35	–	–	23f/13t/5o/6c/1h/3p

### Altered myelin content in patients with TSC and FCD IIB

To explore the differences in myelin pathology among the various forms of MCD, we applied immunohistochemistry to detect the myelin content, using MBP‐, CNPase‐, and MOBP staining. Lesional myelin quantification of the region of interest (ROI) was digitally assessed and compared to autopsy and biopsy white matter. In this and subsequent experiments control white matter was similarly processed.

**Figure 2:**
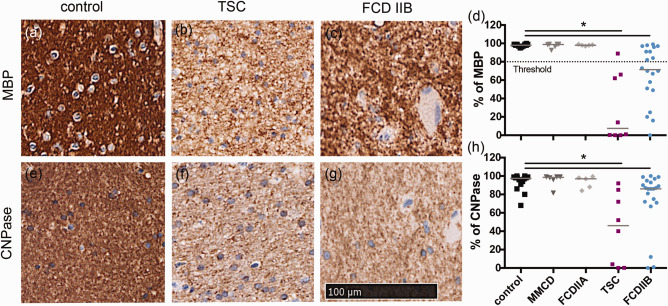
Quantification of myelin pathology shown via % of positive MBP staining in various malformations of the cortical development versus control. Threshold differentiates between normal and hypo‐myelination (80%). **a–c** white matter MBP staining, **d** ROI quantification. **e–g** white matter CNPase staining, **h** quantification. Scale bar = 100 µm counts for all IHC pictures.

The results in Figure 2 showed that using anti‐MBP antibody for detecting myelin, control brain white matter (Figure 2a) stained dark brown compared to white matter obtained from TSC (Figure 2b) or FCD IIB (Figure 2c) patients. Analysis of the intensity of the staining showed that the myelin staining in the control reached a level of 100% (Figure 2d). The lower values for TSC and FCD IIB reached a median level of 7.4% and 71.5%, respectively. The difference was statistically significant (Kruskal–Wallis, pairwise comparisons, *P* = 0.000*, *P* = 0.000*). MMCD and FCD IIA yielded values that resembled those of the control group (Figure 2d). The results using staining with CNPase (Figure 2e–h) and MOBP resembled that with MBP, since statistical analysis revealed a significant reduction in signals in TSC and FCD IIB compared to the control (Kruskal–Wallis, pairwise comparisons: CNPase: *P* = 0.001*, *P* = 0.039*; data not shown MOBP: *P* = 0.003*, *P* = 0.148 n.s.).

As it seems that TSC and FCD IIB lesions were less myelinated compared to other MCDs and control tissue, we examined whether mTOR signaling might have an impact on myelination.

### Association between mTOR activation and lowered myelin content

To explore further the observed myelin pathology and possible associations with mTOR signaling, we analyzed the levels of pS6 representing the downstream activation readout target of mTOR. In addition, we used vimentin as a marker for balloon‐ and giant cells. All positive cells were counted with the automated ROI‐based approach and converted to cells/mm^2^. Myelin densities for MBP, CNPase, and MOBP were analyzed via the intensity of the staining and correlated to the positive cell number.

**Figure 3:**
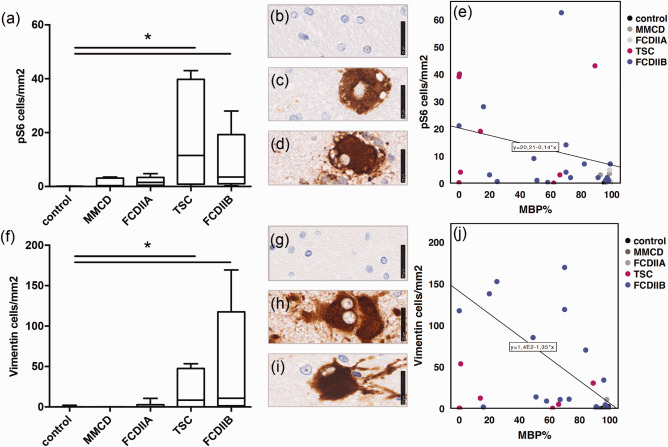
Myelination and mTOR. **a** Cell number from ROI based quantification of pS6. **b** IHC staining of pS6 in control white matter, **c** in balloon cells (FCD IIB), **d** giant cells (TSC), scale bar = 25µm. **e** Correlation of pS6 positive cells and MBP. **f** Cell number from ROI based quantification of vimentin. **g** IHC staining of vimentin in control white matter, **h** in balloon cells (FCD IIB), **i** giant cells (TSC), scale bar = 25 µm. **j** Correlation of vimentin positive cells and MBP.

Our measurements revealed a significant expression of pS6 and vimentin only in TSC and FCD IIB compared to the control (Figure 3a, Kruskal–Wallis, pairwise comparisons, pS6 *P* = 0.009*, *P* = 0.000*; Figure 3f, vimentin *P* = 0.015*, *P* = 0.003*). To correlate these values with myelin abundance, statistical analysis was undertaken, which revealed a significant correlation between increased pS6 expression and lowered myelination (Figure 3e, Kendall‐tau b, MBP: *R* = −0.395, *P* = 0.000*; data not shown‐CNPase: *R* = −0.238, *P* = 0.016*, MOBP: *R* = −0.121, *P* = 0.227 n.s.). Similarly, a significant correlation was also found between higher balloon/giant cell (vimentin) content and decreased myelination (Figure 3j, Kendall‐tau b, MBP: *R* = −0.435, *P* = 0.000*; data not shown‐CNPase: *R* = −0.386, *P* = 0.000*; MOBP: *R* = −0.266, *P* = 0.011*). The blurred grey‐white matter boundaries were detected with NeuN, which correlated with MBP but not with the other myelin markers (Data not shown, Kendall‐tau b, MBP: *R* = −0.231, *P* = 0.04*; CNPase: *R* = −0.64, *P* = 0.567 n.s.; MOBP: *R* = −0.199, *P* = 0.08 n.s.). The inflammatory markers CD3 and CD68 did not show any association with myelination.

Hence, an increased mTOR activation might be linked to a decrease in myelination in TSC and FCD IIB and, therefore, we wanted to measure the effects of this activation on oligodendrocytes.

### Different subpopulations of oligodendroglial cells based on their cell counts

To characterize the cell content and compare it to other malformations of cortical development, we used antibodies against oligodendrocyte transcription factor 2 (Olig2) and analyzed it using the ROI‐based approach. For in‐depth characterization of cell proliferation, antibodies against Ki67 were used in combination with a semi‐quantitative rating technique. To assess whether the visible proliferating cells were indeed oligodendroglia, a co‐localization of fluorescent labeled Olig2/Ki67 antibodies was performed, followed by the same semi‐quantitative analysis.

**Figure 4:**
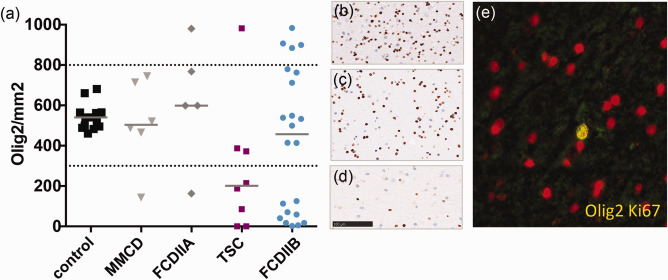
**a** Oligodendrocyte numbers in MCDs. Cell number from ROI based quantification of Olig2. Olig2 subpopulations cluster in low, normal and high cell density. **b** IHC Olig2 staining of “high,” **c** “normal” and **d** “low” levels, scale bar = 100µm. **e** FCD IIB high Olig2 content, IF double staining (yellow) of Ki67 (green) and Olig2 (red) 400×.

Relying on the distribution of frequencies and through unsupervised clustering, we found three subpopulations based on oligodendroglial numbers (Figure 4a–d; threshold = low : max; 300 cells/mm^2^; high : min 800 cells/mm^2^). One subpopulation showed a reduced amount of Olig2 positive cells (Figure 4d) whereas the other an increased oligodendroglial number (Figure 4b) compared to normal levels (Figure 4c). Subsequently, all pathologies were categorized into three groups based on the oligodendroglial content of white matter: low, normal and high. In depth immunohistochemical characterization identified proliferating Ki67 positive cells within the white matter of the “high” group (median = 3%, range = 1%–17%) whereas this was just sporadically observed in the other groups (low : median = 1%, range = 0%–12%; normal: median = 0, range = 0%–1%). Double immunofluorescence staining with Olig2 and Ki67 antibodies showed that a number of dividing cells were oligodendroglia (high: median = 2%, range = 0%–2%; low : median = 0%, range = 0%–1%; normal = 0%, range 0%–1%; Figure 4e). The findings were most prominent in FCD IIB. The eight TSC samples presented similar variations, but ranged between normal and decreased numbers of oligodendroglia compared to controls (Figure 4a).

Since it was possible to detect a subpopulation in FCD IIB, FCD IIA and one TSC with an increased number of oligodendroglial cells, we analyzed whether the cell number might be linked to a better myelination status or to other clinical markers, including seizure frequency or epilepsy duration.

### Link between relative epilepsy duration, mTOR activation, and white matter pathology

Analysis of oligodendroglial cells within the histological white matter lesions of FCD IIB and TSC revealed an inhomogeneous cell content, which could be separated into three populations (Figure 4). To investigate group specific differences, we also sub‐grouped the obtained immunohistochemical data of the myelin stainings (MBP and CNPase) and compared them to mTOR activation and clinical identities. Control staining and data processing were applied using autopsy and biopsy white matter.

**Figure 5:**
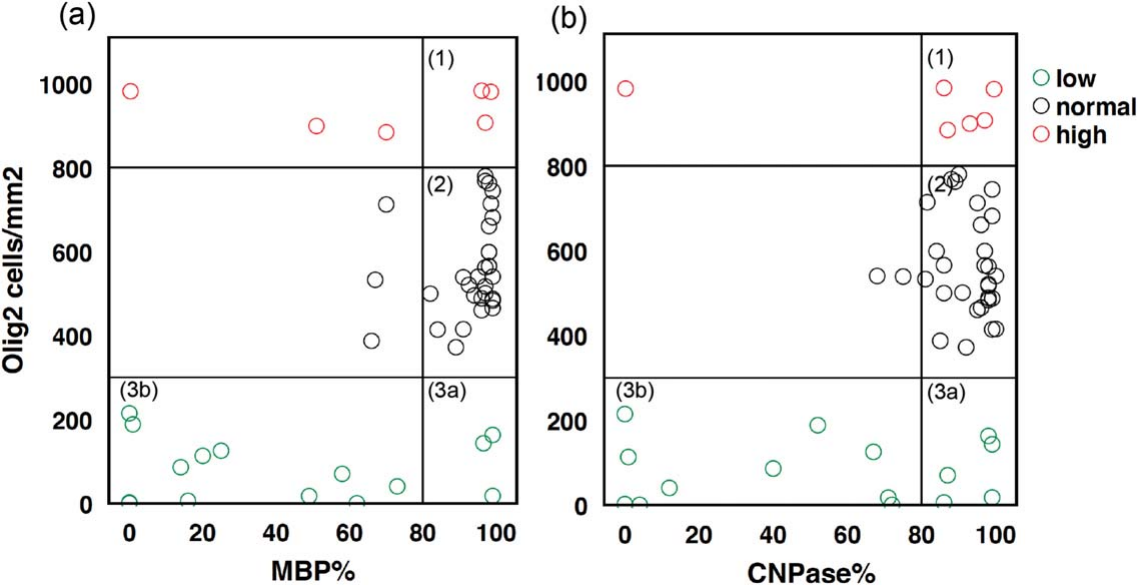
Oligodendrocyte subpopulations in MCDs. Cell number from ROI based quantification of Olig2 and myelin content in %. **a** MBP staining versus Olig2 positive cells/mm^2^. **b** CNPase staining versus Olig2 positive cells/mm2. Sector 1 indicates cases with reactive oligodendroglial hyperplasia and normal myelin content, sector 2 cases with normal myelin and oligodendrocyte numbers and sector 3 burnout of oligodendroglia either with normal myelin levels (3a) or with severe cell and myelin loss (3b).

Further analysis of myelin pathology, shown in Figure 5, evaluated more severe cases within the group with a low content of Olig2 positive cells. CNPase staining showed a median density of 46% (Figure 5b, sector 3b) and MBP a median of 18% (Figure 5a, sector 3b) compared to the normal group (median 99%). The population with a high Olig2 content reached the control group level within both myelin markers (Figure 5a,b sector 1, CNPase median = 90% “high”/91% “normal”; MBP median = 83% “high”/94% “normal”). Analysis of clinical data (Tables 2 and 3), such as cognitive impairment, frequency of seizures, AEDs, and postoperative outcome failed to detect significant group differences. Only the relative duration of epilepsy (normalized to age) correlated to lowered myelination of MBP and CNPase staining (Kendall‐tau b; MBP *R* = −0.325, *P* = 0.002*, CNPase *R* = −0.264, *P* = 0.011; data not shown) and to mTOR activation (Kendall‐tau b; pS6 *R* = 0.338, *P* = 0.001*, vim *R* = 0.274; *P* = 0.011; data not shown).

**Table 3:** Patient medication and outcome. N refers to number of cases. Applied (+) medication were grouped in mTOR inhibitors and anti‐epileptic‐drugs (AEDs): LEV (Levetiracetam), VPA (Valproate), Na^+^ channel blockers, GABA receptor, other AEDs. Postoperative outcome was measured 12 months after surgery (according to Wieser et al. 45). * indicates missing data.

Patient medication and outcome								
Type	*n*	LEV	VPA (−/+)	Na^+^ channel (−/+)	mTOR (−/+)	GABAR (−/+)	other AEDs (−/+)	post OP outcome Wieser (1a/1/2/3/4/5)
TSC	8	1:7	4:4	2:6	6:2	2:6	1:7	3/1/1/2/1/0
MMCD	6	0:6	0:6	0:6	6:0	2:4	1.5	1/1/0/0/1/3
FCD IIA	6	4:2	5:1	0:6	6:0	5:1	3:3	2/1/0/0/1/2
FCD IIB	22	6:13*	7:12*	2:17*	19:0*	8:11*	7:12*	13/4/0/1/0/1*
Total	42	13:28	16:23	4:35	37:2	17:22	12:27	19/8/1/3/3/6

Since it was possible that an increased amount of oligodendroglia might be associated with a better myelination status, we were interested in whether the myelin pathology would be linked to an oligodendroglial renewal problem.

### Burnout of oligodendroglial progenitor cells correlates to lowered oligodendroglial cell numbers

In order to clarify whether a failure within the oligodendroglial lineage might be responsible for the alteration within the myelin pathology, several markers, including PDGFRa, NogoA and Tppp, were tested immunohistochemically and analyzed with the ROI‐based approach. PDGFRa was used to detect early oligodendroglial progenitor cells, NogoA for fully mature cells and Tppp for the oldest ones. To verify the results gained with immunohistochemistry, we obtained western blotting of myelin (MBP), oligodendrocytes (Olig2), and their later precursor cells (NG2), in a small cohort. In detail, three FCD IIB and two TSC white matter lysates were compared to an age‐matched group of two MMCD cases and one non‐epileptical autopsy case.

**Figure 6:**
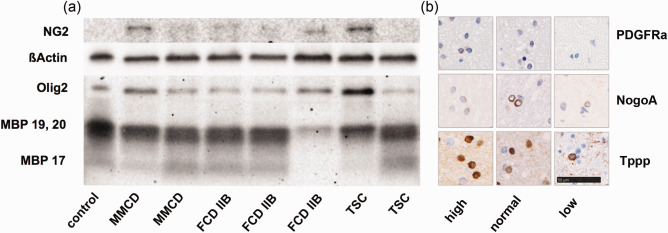
Oligodendroglial lineage markers. **a** Western blot of myelin (MBP isoform 17, 19 and 20), oligodendrocytes (Olig2) and their precursors cells (NG2) in MMCD, FCD IIB and TSC white matter samples compared to one autopsy control. ßActin was used as loading control. **b** IHC staining of old (Tppp), mature (NogoA) and juvenile (PDGFRa) oligodendroglia in representative white matter lesions of low, normal and high Olig2 populations. Scale bar = 50 µm, counts for all IHC pictures.

The immunohistochemistry results showed that mature oligodendroglial indicators like NogoA and Tppp were present in all groups (Figure 6b; Kruskal–Wallis; no significant differences). In contrast, only the early OPC marker PDGFRa correlated to the overall number of oligodendroglia (Figure 6b, Kendall‐tau b; Olig2 *R* = 0.309, *P* = 0.030*). Protein detected using western blotting revealed ranging conditions within oligodendroglia. Myelin content was lowered in one TSC and one FCD IIB case (Figure 6a). Interestingly, NG2‐precursor cell activation was connected to MBP 17 isoform loss.

Hence, myelin loss in combination with a lowered amount of oligodendroglia was only seen in TSC and FCD IIB, and we discuss below the possible impairment in the development of oligodendroglial progenitor cells into myelinating oligodendrocytes.

## Discussion

In the present study, we generated large‐scale data on the myelination status in malformations of cortical development (MCD). The use of a region of interest (ROI)‐based approach was shown to be efficient and reliable for data collection and analysis. Using ROIs, we were able to show for the first time that FCD IIB and TSC brains contained axons with less myelination compared to age and region matched healthy white matter. In addition, we were able to detect a novel lesion‐specific mTOR activation. The lowered myelin content correlated with increased mTOR expression and the severe white matter pathology was further linked to the relative duration of epilepsy. We also identified for the first time two oligodendroglial subpopulations in patients with MCD, based on reduced numbers of oligodendroglial cells and proliferative oligodendroglial contents. Moreover, immunohistochemistry evaluation of oligodendroglial precursor cells revealed that adequate numbers of PDGFRa positive cells correlated with the amount of oligodendroglia. Western blotting showed that in those groups with specific myelin loss (MBP isoform 17), NG2 positive cells were activated, indicating delayed development into mature myelinating oligodendrocytes.

Recently, two rather arbitrary studies regarding hypomyelination and the numbers of oligodendroglial cells in epilepsy surgery specimens have been published 23, 35. Whereas the first study showed that myelination was exclusively reduced in FCD IIB and correlated with a reduced number of oligodendroglial cells, the group of Shepherd *et al*, was not able to identify significant differences within oligodendroglia or OPC counts. Furthermore, in 2015 Zucca *et al* 49 were able to highlight lower amounts of oligodendroglia. In our current study, we were also able to detect a lower amount of oligodendroglial cells in a subpopulation of FCD IIB, albeit not exclusively. We believe that the findings of our current study reflect a more continuous spectrum of white‐matter pathology also in FCD IIB, which might close the gap between the two previously published contradictory results published so far. Therefore in this study, we focused on causative factors: for example, OPCs correlate with oligodendroglia, relation to mTOR activation, and the proliferative capabilities of oligodendroglia. Additionally, we expanded the analyzed cohort and included also TSC samples, which had not been analyzed in previous studies.

The present results were based on quantitative immunohistochemistry, which enabled us to detect very slight differences between patient subgroups. The ROI approach employed here had the benefit of exploiting the whole pathologic region for analysis, which was kept constant during the different staining procedures compared to other reposts where one single square of an individually selected part of the lesion was used for analysis 34, 35. To avoid additional bias from intra and interpersonal evaluation methods, standardization of the quantitative picture analysis was undertaken using a dedicated ImageJ macro specifically designed to allow full automatic processing of region‐specific stainings. With this method, we evaluated the extent of myelin pathology of TSC tubers and FCD IIB compared to other MCDs. It was possible to demonstrate a significant reduction within MBP and CNPase in 62% of TSC and 50% of FCD IIB patients, while other MCDs showed no alterations. This was in line with previous studies that reported similar results 23, 35, 49. It was also shown previously that myelin‐related mRNA transcripts are reduced in the lesional cortex of FCD patients 10.

Our study provides evidence of an association between the myelin pathology and deregulation of the mTOR pathway. A number of studies have been performed investigating the role of the AKT/mTOR pathway in myelination [recently reviewed in Ref. ( 11)]. Many of these studies used rapamycin to inhibit mTOR function and subsequently examined the function of oligodendrocytes that were less capable of producing sufficient myelin 46. However, there is increasing evidence that mTOR signaling plays a crucial role in oligodendroglial differentiation and myelination in a time‐dependent manner 11. In contrast, a large volume of evidence suggests that myelination deficits may occur in conditions with constitutive mTOR activation, such as TSC, and is also related to cognitive deficits and autism although the underlying mechanisms remain unclear 29, 37. Very recently, it has been shown that loss of TSC2 in oligodendrocytes leads to oligodendroglial malfunction and impaired communication with neurons 7. This is in line with our finding that further supports the pathogenic link between mTOR activation and myelination deficits.

For TSC patients, an association between the TSC1/2 mutation and the mTOR pathway is well described 20. TSC is naturally a mutation of either TSC1 or TSC2 in 85%, whereas more than half of those cases show also mosaicism 43. Recent studies found somatic (mosaic) mutations affecting the mTOR signaling also in focal lesions of FCD II patients 4, 8, 21, 25, 32, 36. This is a very important aspect since as far as we know, in FCD, the mutations are localized and show profound variability in cellular expression. Thus, mosaicism seems to play an important role in these lesions. However, in most FCDs, we are just analyzing the epileptogenic lesion where seizures have their origin. In terms of genetic disposition, it is definitely possible that the mosaic mutations occur also in other brain regions, but the fraction of mutated cells is not big enough to trigger malformation. Therefore, we sequenced the DNA of the lesional tissue of 22 samples of our FCD IIB cohort for the presence of validated epilepsy associated mutations, but could only detect mutations in only three patients. However, it remains possible that the other patients had mutations within different genomic regions or related genes, which might turn out to play a major role in disease development.

White‐matter deficiencies are strongly associated with missing myelin‐producing cells and/or glial malfunction 5, 49. However, the number of oligodendroglial cells in MCDs is the subject of ongoing debate 12, 23, 35. We showed for the first time that the number of oligodendroglial cells may vary from case to case even within the same type of lesion (tubers, FCD IIB). On the one hand, there was a reduced amount of cells in the case of lower myelination, as described previously 24, 49, but on the other hand we were able to show that an increased proliferative population of oligodendroglia lead to a better myelination status. Within this context, based on our results we suggest that severe myelin deficits can indeed be associated with a decrease in Olig2‐positive cells. However, normal to high amounts of oligodendroglia were previously found in normal myelination. Indeed, in patients with temporal lobe epilepsy and MMCD it has been shown before that proliferative oligodendroglial hyperplasia has no negative impact on myelination 34, 40. In our cohort, we identified proliferative oligodendroglia for the first time also in FCD IIA, IIB, and TSC, indicating a reactive phenomenon.

Finally, we investigated oligodendroglial lineage maturation in order to clarify the mechanisms of demyelination. Compared with a multi‐focal demyelinating disease such as MS, we were unable to detect signs of remyelination by immunhistochemistry. Nonetheless, also in chronic MS there is not only a decrease in the number of oligodendrocytes but also a decrease in the capacity for maturation in the remaining oligodendroglial cells 30. Yet, failure of cortical remyelination in chronic MS may not simply result from repeated episodes of cortical demyelination affecting mature oligodendroglia, but may also require other factors that would damage OPCs at an early differentiation stage 31. In our epilepsy cases, missing early OPCs (PDGFRa+), activation of later OPCs (NG2+), and an overall lower content within mature oligodendroglia, led us to the conclusion that there is a cell breakdown (or burnout) because of insufficient maturation that prevents adequate myelination.

To conclude, here we present the first evidence that inhibition of oligodendroglial cell maturation, presumably because of overtly active mTOR signaling, may contribute to insufficient myelination associated with TSC and FCD IIB. In terms of disease management, it might be beneficial for patients to reduce the activation of mTOR signaling. Since hypo‐myelination seemed to play an additional key role in disease progress, the possibility of reversing myelin disappearance could be another potential approach for treatment. This issue has not been studied so far in TSC and FCD IIB. However, previous studies in patients with MS showed that re‐myelination was possible, and an *in vivo* study conducted on rodent models of MS revealed that certain pharmaceuticals could actively support this process 9. Assuming that hypomyelination is a reversible process, this might enable an adjuvant or even a new, targeted therapy option for patients in a selected population of TSC‐ and FCD IIB‐associated epilepsies.
